# Thrombomodulin Serum Levels—A Predictable Biomarker for the Acute Onset of Ischemic Stroke

**DOI:** 10.3390/cimb46010044

**Published:** 2024-01-12

**Authors:** Andrei-Lucian Zaharia, Dana Tutunaru, Violeta Diana Oprea, Claudiu Elisei Tănase, Ana Croitoru, Bianca Stan, Doina Carina Voinescu, Ana-Maria Ionescu, Camelia Alexandra Coadǎ, Mihaiela Lungu

**Affiliations:** 1 “St. Apostle Andrei” Clinical Emergency County Hospital Galati, 800578 Galati, Romania; zaharia.andreilucian@gmail.com (A.-L.Z.); croitoruana28@yahoo.com (A.C.); csb.bianca@gmail.com (B.S.); carinavoinescu@gmail.com (D.C.V.); mihaelalungu17@yahoo.com (M.L.); 2 Faculty of Medicine and Pharmacy, “Dunarea de Jos” University of Galati, 800216 Galati, Romania; tanaseclaudiumd@gmail.com; 3 “St. Joan” Pediatric Clinical Emergency Hospital Galati, 800487 Galati, Romania; 4 Faculty of Medicine and Pharmacy, Ovidius University of Constanța, 900470 Constanța, Romania; iuliusana@gmail.com; 5 Faculty of Medicine, “Iuliu Hatieganu” University of Medicine and Pharmacy, 400012 Cluj-Napoca, Romania; coada_camelia_alexandra@elearn.umfcluj.ro

**Keywords:** thrombomodulin, acute ischemic stroke, biomarker, thrombolysis, early diagnosis

## Abstract

The early diagnosis of acute ischemic stroke (AIS) can be challenging in cases presenting with a scarcity of clinical signs, normal cerebral imaging in early stages and a lack of specific serum markers. Thrombomodulin has been shown to be associated with cerebrovascular ischemic events and can be considered an important biomarker for the acute onset of ischemic stroke. In our study, we compared the serum levels of thrombomodulin (sTM) between a relevant patient group of 70 AIS patients and a control group of patients without AIS admitted into the neurology department between June 2022 and May 2023. sTM levels were measured at 24 h and 48 h after patients’ admissions into the hospital. There was a significant difference between the two groups (AIS: 23.2 ± 9.17 ng/mL vs. controls: 3.64 ± 1.72 ng/mL; *p*-value < 0.001). sTM values were correlated with the score of neurological deficits, with gender and dyslipidemia. The association of sTM values with the acute onset of AIS as an end point was significant, which allows rapid therapeutic interventions, even in the absence of a well-defined clinical syndrome (AUC = 0.99). Reanalysis of the patients after propensity score matching increased the power of sTM as a biomarker (AUC = 1). sTM represents a potentially useful biomarker to diagnose the onset of an AIS, even in scarce clinical presentations, which makes thrombomodulin a valuable indicator for early treatment initiation.

## 1. Introduction

Acute ischemic stroke (AIS) is one of the leading causes of morbidity and mortality worldwide. The diagnosis of acute AIS can be challenging, as clinical signs are occasionally inconclusive [1]. Computed tomography (CT) may appear normal in the early stages of AIS or in patients with minor symptoms or with strokes in the vertebrobasilar system. Since emergency imaging investigations are not always available, many blood markers have been proposed for the diagnosis of stroke in the acute setting. The availability of a rapid test to confirm a clinical or imaging diagnosis of AIS or to quantify the risks in such situations would be extremely useful, as it would allow more patients to benefit from the timely administration of thrombolysis. Currently, the diagnosis of AIS is based on the clinical experience of the neurologist, aided by neuroimaging [2]. However, in patients who unexpectedly develop a malaise suggestive of AIS, clinical assessment in the first few hours is not always sufficient. Establishing an early diagnosis of AIS is essential for the rapid initiation of appropriate treatment (thrombolysis, thrombectomy), as well as implementing secondary prevention [3]. This urgency is underscored by the fact that 8% of high-risk patients have a recurrent stroke within the initial 2 days [2,4]. The extent of brain lesions and their instability in the absence of early treatment represent unfavorable prognostic elements in AIS. Many potential blood biomarkers of cerebral ischemia and inflammation are found in other conditions that may mimic a stroke, such as myocardial infarction and brain infections. Also, the volume of brain damage by ischemia may not correlate with the clinical impact, so that cerebral infarcts of small dimensions located in strategic areas can lead to severe disabilities, compared to extensive lesions that are located in areas with lower clinical impact. Therefore, there is an increased interest in determining new biomarkers for the rapid diagnosis of AIS, as well as the contribution of biomarkers in transient ischemic attack (TIA) [3].

The development of blood biomarkers for AIS is challenging. The blood–brain barrier slows the release of brain tissue proteins into blood immediately after a stroke, delaying the release of glial and neural proteins. Thus, the use of such biomarkers should be considered in cases where the diagnosis of AIS is uncertain [5].

One potential biomarker for stroke is thrombomodulin. Thrombomodulin (TM) is a type-1 transmembrane glycoprotein with primary expression in endothelial cells that plays an important role in a multitude of processes, with biological functions being attributed to various subdomains of soluble TM [6,7].

In the presence of cytokines, activated macrophages and neutrophils, endothelial TM is enzymatically cleaved, releasing soluble fragments that circulate in the blood and are eliminated in the urine. Circulating forms of TM are also present in synovial fluids. Soluble TM (sTM) contains several domains of TM and is the major circulating TM, generated by either enzymatic or chemical cleavage of the intact protein under different conditions. Under normal conditions, sTM is present in low concentrations, below 10 ng/mL in the blood, but it increases in pathological conditions associated with endothelial dysfunction: cardiovascular and inflammatory diseases, infections, and metabolic diseases [8,9]. This suggested that sTM should be monitored as a marker for conditions like intravascular coagulation, sepsis and multiple organ dysfunction syndrome in patients with COVID-19 [7,8,9].

Overall, the plasma level of TM (pTM) can be regarded as a molecular marker reflecting endothelial alteration [10]. TM is often increased in the case of diffuse endothelial destruction, as for example in disseminated intravascular coagulation, microangiopathy, rickettsial infections, neoplastic diseases, systemic lupus erythematosus or atherosclerosis, but it can also be a predictive marker in situations in which the serum level of pTM can be correlated with disease activity [8,9,10].

TM is also found in other cell types, such as keratinocytes, osteoblasts, macrophages, platelets, monocytes and mesothelial cells, where it appears to be involved in cell differentiation or inflammation [11]. TM acts as a cofactor of thrombin in the activation process of protein C, which, via the proteolytic inactivation of activated factors V and VIII, has an anticoagulant role. TM is involved in a compensation mechanism that stops the coagulation cascade and avoids the occurrence of thrombotic events. Moreover, it promotes fibrinolysis through the proteolytic inactivation of plasminogen activator inhibitor (PAI), helping the remodeling of the secondary clot [10,12,13]. Apart from being part of the protein C anticoagulant system, TM has been discovered to interfere with inflammation, stabilize barrier function and increase blood flow under pathological conditions [10,14,15]. Moreover, the administration of recombinant soluble TM has been shown to protect against tissue damage and partially restore normal functions after ischemia in several organs [9,10].

The aim of this work was to evaluate the performance of TM as a differential diagnosis biomarker in patients presenting at the emergency unit with clinical signs of AIS.

## 2. Materials and Methods

### 2.1. Study Design and Patients’ Inclusion Criteria

This was an observational, prospective, analytical monocentric study that enrolled consecutive patients with clinical signs of ischemic stroke in the previous 24 h before hospital presentation and a control group of patients, free from cerebral ischemic context, admitted to the Emergency Clinical Hospital of Galati—Clinical Neurological Department between January 2022 and May 2023. The study was conducted in accordance with the 1964 Helsinki Declaration and was approved by the local Hospital Ethics Committee number 524/07.01.2021.

Inclusion criteria were set as follows: clinical signs and symptoms suggestive of an acute stroke; brain CT scan performed within 24 h after hospital admission and negative for AIS changes (ASPECTS score of 10) [16]; age above 18 years old; existing written consent from patient or family member to be included in the study.

Exclusion criteria were: patients with hemorrhagic stroke, acute myocardial infarction or other neurological pathologies, infections/septic conditions, severe renal or hepatic insufficiency, malignancies, recent surgery or trauma within the last 6 months.

Detailed medical history and clinical data were collected from all patients and/or family members. Head and neck CT scans were performed using the standard protocols including native and contrast-enhanced sequences. All images were stored using the DICOM format. Expert radiologists with >10 years of medical experience analyzed the images looking for any signs of stroke. The National Institutes of Health Stroke Scale (NIHSS) was recorded for all patients by a stroke neurologist, at presentation, after 48 h and at patient discharge. The Trial of Org 10172 in Acute Stroke Treatment (TOAST) classification system was used to categorize the subtypes of AIS [17].

### 2.2. Blood Work and TM Analysis 

All patients had routine screening blood samples, collected immediately after the presentation at the emergency unit. A sample of blood was collected in BD-vacutainers serum tubes without anticoagulants for the dosage of TM at presentation (T_1_) and after 48 h (T_2_). Samples were frozen at −20 °C until analysis. Serum TM levels were measured by the ELISA method, using the HUMAN TM ELISA kit (catalog number E-EL-H0166, provided by ELABSCIENCE (Houston, TX, USA)). The micro ELISA plate was pre-coated with an antibody specific to human TM, with standards or samples added to the wells and combined with the specific antibody. For the analysis, a biotinylated detection antibody specific to human TM and avidin-horseradish peroxidase (HRP) conjugate were added successively to each microplate well and incubated. Free components were washed away. The substrate solution was then added to each well. Only those wells that contained human TM, biotinylated detection antibody and avidin-HRP conjugate appeared blue in color. The enzyme–substrate reaction was terminated by the addition of a stop solution and the color turned yellow. The optical density (OD) was measured spectrophotometrically at a wavelength of 450 nm ± 2 nm, the OD value being proportional to the concentration of human TM. The concentration of human TM in the samples was calculated by comparing the OD of the samples to the standard curve provided by the manufacturer.

### 2.3. Statistical Analysis 

Statistical analysis was performed in R version 4.3.2 [18] (Vienna, Austria). Summary statistics were conducted for all analyzed variables. Continuous variables were reported as median and 25–75 percentiles or mean ± standard deviation while categorical variables were reported as frequency and percentage. Differences between patients’ groups were analyzed using a *t*-test for continuous variables and a Chi-square test for categorical ones. The performance of the binary classification model, employing TM as a diagnostic biomarker, was assessed through the analysis of ROC curves. Propensity score matching was performed using the MatchIt R package (Version 4.5.5) [19]. A *p*-value of ≤0.05 was considered statistically significant.

## 3. Results

### 3.1. Population Characteristics 

**Figure 1:**
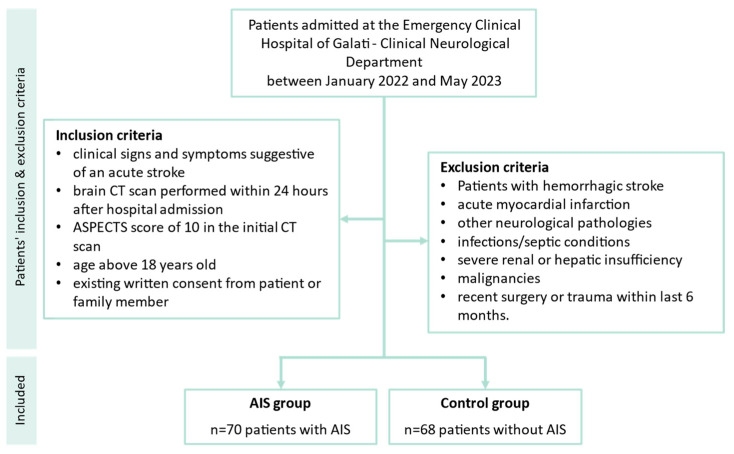
Flow chart presenting the selection process of the patients and controls included in this study. AIS: acute ischemic stroke.

The study included 70 patients diagnosed with acute ischemic stroke and 68 control patients with other non-ischemic pathologies (Figure 1). AIS patients were admitted at the emergency unit for signs and symptoms suggestive of acute stroke. General demographic and clinical characteristics of the study cohort are reported in Table 1. There were no significant differences between the study groups in terms of age, sex and residential background. There were significant differences between the two groups in terms of comorbidities (diabetes, hypertension) as well as chronic alcohol and tobacco use (Table 1).

Regarding the type of stroke, most patients (43, 61.43%) had an atherothrombotic event, while the remaining 27 (38.57%) patients presented with a cardioembolic stroke (Table 2). The most frequently affected vascular territory was represented by the left middle cerebral artery in 32 (45.71%) cases followed by the right middle cerebral artery in 24 (34.29%) cases and the vertebrobasilar system in 14 (20%) cases (Table 2). Eight (11.43%) patients died after hospital admission. 

### 3.2. Serum TM Levels Significantly Increase in Acute Stroke Patients

**Figure 2:**
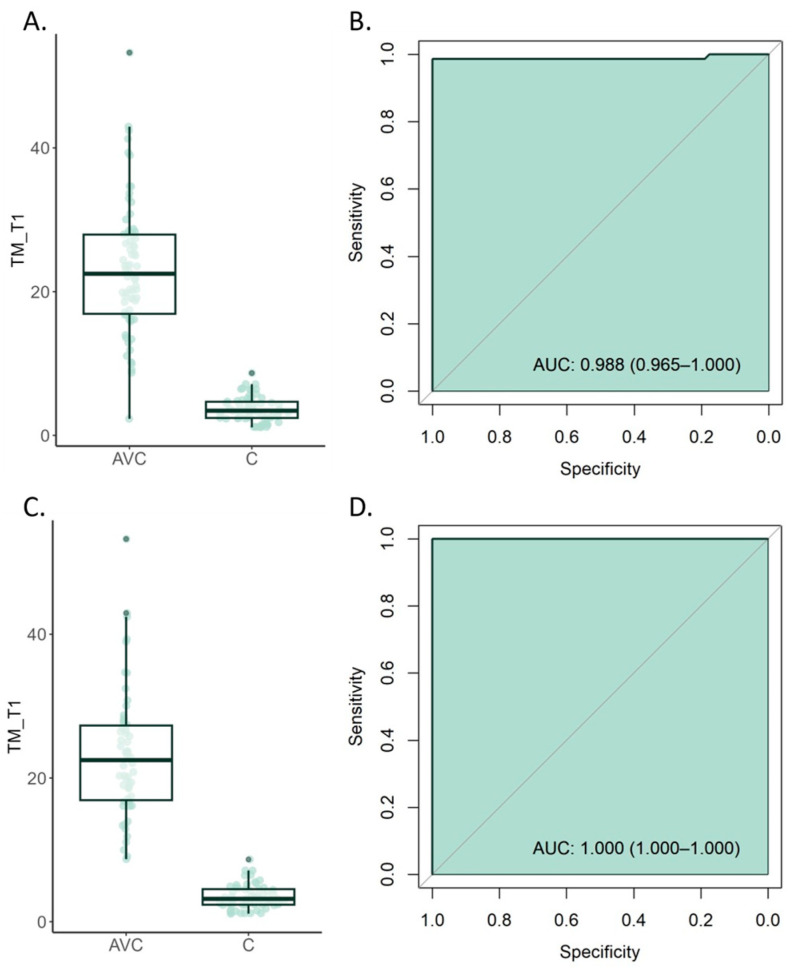
(**A**) Comparative analysis of serum TM levels in patients with acute stroke (study group) and controls. (**B**) Receiver operating characteristic (ROC) curve depicting the area under the curve (AUC) for serum TM measurements within the initial 24 h of hospitalization. (**C**,**D**) The figures illustrate the same analyses after the popensity score matching.

Comparison between stroke patients and those without stroke revealed a significant increase in TM levels in the blood of stroke-affected individuals. Specifically, stroke patients exhibited an average TM concentration of 23.2 ± 9.17 ng/mL, whereas control patients demonstrated a markedly lower level at 3.64 ± 1.72 ng/mL (*p*-value < 0.001) (Figure 2A). 

We further conducted an ROC analysis to assess the efficacy of TM as an early biomarker for the identification of AIS patients. Our findings reveal that, at a serum threshold of 8.69 ng/mL, TM presented a highly differentiating capability in diagnosing stroke among clinically symptomatic patients, achieving an accuracy of 0.99 (Table 3). The specificity was calculated as 1, while the sensitivity was 0.98. Moreover, the positive predictive value (PPV) and negative predictive value (NPV) were computed as 1 and 0.98, respectively (Figure 2B, Table 3). 

Given the presence of differences in comorbidities and patient history (Table 1), we performed a propensity score matching to mitigate any possible biases caused by these variations. Thus, we constructed a model that incorporated the following variables: diabetes, hypertension, chronic alcohol and tobacco use, to select a well-matched subgroup of patients. Reanalysis of this matched subgroup (N = 58 cases/each group) showed that the propensity score matching successfully eliminated significant differences among the study participants (Supplementary Figure S1; Supplementary Table S1). Reanalysis of the levels of TM confirmed our previous results. Namely, after propensity score matching, the stroke group showed significantly elevated TM blood levels compared to their counterparts in the control group (23.19 ± 8.91 ng/mL vs. 3.5 ± 1.7 ng/mL; *p*-value < 0.001) (Figure 2C). Moreover, the ROC analysis demonstrated an exceptional AUC value of 1 (Figure 2D, Table 3).

### 3.3. Serum TM Levels Correlation with Clinical Features in Patients with Developing Stroke

Serum TM levels were assessed at two distinct time points: at patient presentation and 48 h thereafter. A comparison between these measurements revealed stability in TM levels, at least within the initial 48 h following the stroke (23.2 ± 9.17 vs. 23.09 ± 8.34; *p* = 0.361). TM levels at T_1_ were found to be higher in stroke patients with dyslipidemia than in those without (25.12 ± 9.64 vs. 20.14 ± 7.57; *p* = 0.021) (Supplementary Table S2). Moreover, TM levels correlated with LDL cholesterol and total cholesterol as well as liver transaminases (Supplementary Table S5, Supplementary Figure S2). Male stroke patients had higher TM values than females at both measured timepoints (T_1_: 24.87 ± 9.42 ng/mL vs. 20.53 ± 8.25 ng/mL; *p* = 0.053 and T_2_: 25.11 ± 8.76 ng/mL vs. 19.82 ± 6.53 ng/mL; *p* = 0.01) (Supplementary Tables S2 and S3). 

We sought to evaluate the correlation between serum TM levels and the specific type of stroke. When comparing patients with cardioembolic strokes to those with atherothrombotic strokes, our analysis revealed no significant correlation between TM levels at both the initial measurement time point (T_1_) (21.55 ± 9.3 ng/mL vs. 24.23 ± 9.05 ng/mL; *p* = 0.236) and the subsequent time point (T_2_) (22.66 ± 8.18 ng/mL vs. 23.35 ± 8.53 ng/mL; *p* = 0.744) (Supplementary Table S4). 

We also performed a comparative analysis between patients who underwent thrombolysis as part of their stroke treatment and those who did not. We found slightly lower serum levels of TM in patients receiving thrombolysis compared to those without, both at T_1_ (19.16 ± 6.7 ng/mL vs. 23.87 ± 9.4 ng/mL) and T_2_ (19.33 ± 7 ng/mL vs. 23.74 ± 8.43 ng/mL), albeit these differences did not achieve statistical significance (*p* = 0.134 and *p* = 0.124, respectively). 

**Figure 3:**
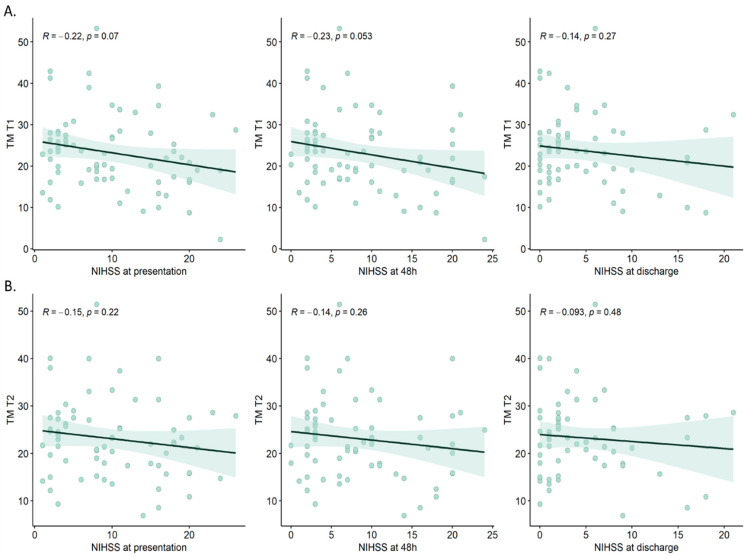
Correlation analysis between serum TM levels at T_1_ (**A**) and at T_2_ (**B**), and NIHSS measured at patient presentation, 48 h and at discharge. R: Pearson correlation coefficient.

Next, we aimed to assess the correlation between serum TM levels and the severity of stroke, as measured by the NIHSS. The results reveal a modest correlation between TM levels and NIHSS at the time of presentation (Pearson coefficient = −0.22, *p* = 0.07). Similar findings were observed in the correlation analysis conducted with the NIHSS at 48 h post-presentation (Pearson coefficient = −0.23, *p* = 0.053). Notably, the strength of the correlation appeared to diminish with the NIHSS at the time of discharge (Pearson coefficient = −0.14, *p* = 0.27) (Figure 3A). Similarly, this correlation was reduced when analyzing serum TM levels at T_2_ (Figure 3B). 

## 4. Discussion

In recent studies, TM has been shown to reduce cerebral infarct size in stroke models [14,20]. Compared to other anticoagulants, the risk of bleeding appears to be lower in animals and humans treated with sTM, suggesting its protective role in preventing brain damage in stroke [20,21].

TM has been a subject of interest in multiple studies tackling its role in various diseases. It has been shown that an increased level of sTM can correlate with cardiovascular diseases, intravascular coagulation, multiorgan failure or death as well as ischemic stroke [22,23]. In healthy individuals, the level of sTM is below 10 ng/mL, while elevated values of sTM are found in patients with various conditions [20,24,25].

As such, we initiated our study from the hypothesis that measuring the serum level of TM might be considered as an early diagnostic tool of endothelial destruction in ischemic stroke at onset.

A study also revealed the correlation between serum TM and the risk of atherosclerotic disease [26]. Recent studies have shown that the level of sTM is significantly increased in patients with atherosclerosis lesions versus healthy individuals, with the level directly correlating with the number of lesions [23,25,27]. Atherosclerosis is one of the risk factors of AIS. Our results show an association between TM levels and dyslipidemia in the AIS patients included in this study. However, we found no association between TM levels and the type of AIS.

Zhu et al. conducted a multicenter prognostic cohort study of 3532 Chinese ischemic stroke patients, concluding that increased plasma TM levels at baseline were associated with decreased risks of adverse clinical outcomes at 3 months after ischemic stroke, suggesting a protective role of thrombomodulin in the development of ischemic stroke [20]. Although we did not measure the long-term clinical outcomes of our patients, short-term follow-up revealed an inverse correlation between TM levels and the severity of AIS. This result might suggest that a significant rapid increase in TM immediately during the initial phase of the stroke development could contribute to the limitation of the damage. This hypothesis is supported by works showing that the administration of recombinant soluble TM (sTM) protects against tissue damage and partially restores normal function after ischemia in several organs. Recently, studies demonstrated that sTM reduces the infarct size in stroke models. Compared to other anticoagulant compounds, the risk of bleeding seems to be smaller in animals and humans treated with sTM. With its multiple actions, some researchers consider that TM represents a new candidate for stroke treatment [6,21]. The foundation of this crucial protective role lies in the mechanism through which TM regulates anticoagulant activity. TM not only inhibits thrombin, but also decreases the affinity of procoagulant substances at the level of the infarct zone. TM directly inhibits the procoagulant functions of thrombin, fibrinogen, platelet aggregation and factor V Leiden [25,26,27,28,29] and accelerates the inactivation and degradation of thrombin by inhibiting both antithrombin and C protein [28,29,30,31].

In our study, we sought to explore the diagnostic potential of TM. We found that patients with a TM value exceeding 8.69 ng/mL were confirmed to have a stroke. Moreover, given all the variations of TM in different diseases, we conducted a propensity score matching to minimize any biases caused by the patients’ comorbidities, which resulted in significant differences among the study groups. After the propensity score, we obtained an excellent AUC of 1. This robust result underscores the precision and accuracy of TM as a biomarker for distinguishing between the AIS and control patients, providing clinicians with a highly reliable tool for the timely and accurate identification of stroke cases.

The importance of patients’ comorbidities and concomitant diseases, such as cancer, was elegantly explored in the Mechanisms of Ischemic Stroke in Cancer (MOST-Cancer) study. The study showed that patients with cancer and AIS have higher serum markers of coagulation, platelet and endothelial activation, and more circulating microemboli than patients with cancer only or AIS only [32]. Of all the biomarkers analyzed among cancer-plus-stroke participants, it was noted that only D-dimer, *p*-selectin, sICAM-1, sVCAM-1 and microemboli were associated with the primary outcome (composite of major thromboembolic events or death), whereas thrombin-antithrombin and TM were not. D-dimer was the only marker associated with recurrent AIS.

It is worth discussing the contrasting observation that populations with genetically determined elevated pTM levels appear to experience worse long-term vascular status, leading to increased risks of ischemic stroke and adverse clinical outcomes [14]. Hongzhou et al. conducted a two-sample mendelian randomized study and demonstrated that a genetic predisposition to elevated pTM levels was associated with an increased risk of ischemic stroke [20]. This appears to contradict the body of research showing that elevated TM levels in AIS can play a protective role. These discrepancies show that different potential mechanisms for elevated pTM may lead to dissimilar outcomes. For example, a previous observational study revealed that TM reduced the primary risk of brain infarction in patients without previous vascular history and, conversely, it increased the death rate in patients with brain infarction [24]. Furthermore, the relationship between sTM levels and mortality after stroke was confirmed in patients in the National Institute of Neurological Disorders and Stroke (NINDS) rtPA Stroke Study [33]. Our analysis did not find a significant association between TM levels and death. This might be due to the fact that only a small proportion of patients died during the hospitalization time. It is also worth mentioning that we had only one patient with a previous history of AIS, which seems to be the scenario where TM is of predictive value.

The limitations of our analysis are related to the relatively small patient group, the single-center study and the short follow-up period, which could potentially bring further relevant data regarding the prognostic potential of TM in AIS. The strengths of our study are represented by the homogenous cohort of patients in terms of explorations and treatment, as they were managed by the same experienced medical team. Moreover, the propensity matching selected a subgroup of patients with a similar distribution of comorbidities, further consolidating our results. Next, the repeated measurements of TM at a distant timepoint showed the reliability of the test due to its consistent levels.

## 5. Conclusions

Serum TM levels may represent a potentially valuable biomarker for the diagnosis of the onset of an AIS, even with scarce clinical neurological manifestations, positioning TM as an alarm signal for treatment initiation. AIS patients presented a significant increase in TM values in the first 24 h after onset.

Moreover, serum levels correlated with the severity of AIS development and the risk of death, being influenced by associated cardiovascular risk factors. Patients receiving thrombolysis did not influence the levels of TM at T_1_ nor at T_2_.

Further research and measurement standardization are necessary to exactly determine the value of pTM level assessment in AIS and its role as a diagnostic and predictive biomarker.

## Figures and Tables

**Table 1:** Demographic, clinical and pathological profiles of patients diagnosed with acute stroke and control group. N: number of patients; SD: standard deviation.

Variable		Control Group N = 68	Stroke Group N = 70	*p*-Value
Age (years) mean ± SD		69.28 ± 10.5	70.24 ± 10.93	0.598
Gender N (%)	Female	30 (44.12)	27 (38.57)	0.508
	Male	38 (55.88)	43 (61.43)	
Residential background N (%)	Urban	36 (52.94)	46 (65.71)	0.127
	Rural	32 (47.06)	24 (34.29)	
Atrial Fibrillation N (%)	No	51 (75)	43 (61.43)	0.087
	Yes	17 (25)	27 (38.57)	
Dyslipidemia N (%)	No	34 (50)	27 (38.57)	0.177
	Yes	34 (50)	43 (61.43)	
Diabetes N (%)	No	41 (60.29)	55 (78.57)	0.020
	Yes	27 (39.71)	15 (21.43)	
Hypertension N (%)	grade 1	11 (16.18)	5 (7.14)	0.027
	grade 2	32 (47.06)	24 (34.29)	
	grade 3	25 (36.76)	41 (58.57)	
Chronic Alcohol Use N (%)	No	47 (69.12)	60 (85.71)	0.020
	Yes	21 (30.88)	10 (14.29)	
Chronic Smoker N (%)	No	45 (66.18)	60 (85.71)	0.007
	Yes	23 (33.82)	10 (14.29)	

**Table 2:** Clinical characteristics and laboratory parameters of the patients with acute stroke included in this study. N: number of patients; sd: standard deviation; NIHSS: National Institutes of Health Stroke Scale; HDL: high-density lipoprotein; LDL: low-density lipoprotein.

Variable		N (%)/Mean ± SD
Clinical features		
Stroke type N (%)	Cardioembolic	27 (38.57)
	Atherothrombotic	43 (61.43)
Affected vascular territory N (%)	Left middle cerebral artery	32 (45.71)
	Right middle cerebral artery	24 (34.29)
	Vertebrobasilar system	14 (20)
Received thrombolysis	No	60 (85.72)
	Yes	10 (14.28)
Exitus N (%)	No	62 (88.57)
	Yes	8 (11.43)
NIHSS at presentation mean ± SD		10.07 ± 6.93
Severity of NIHSS at presentation N (%)	minor (NIHSS = 0–4)	22 (31.43)
	moderate (NIHSS = 5–15)	28 (40)
	moderate to severe (NIHSS = 16–20)	15 (21.43)
	severe (NIHSS = 21–42)	5 (7.14)
NIHSS at 48 h mean ± SD		8.53 ± 6.67
Severity of NIHSS at 48 h N (%)	no stroke signs (NIHSS = 0)	2 (2.86)
	minor (NIHSS = 0–4)	26 (37.14)
	moderate (NIHSS = 5–15)	27 (38.57)
	moderate to severe (NIHSS = 16–20)	12 (17.14)
	severe (NIHSS = 21–42)	3 (4.29)
Severity of NIHSS at discharge N (%)	no stroke signs (NIHSS = 0)	12 (19.35)
	minor (NIHSS = 0–4)	27 (43.55)
	moderate (NIHSS = 5–15)	17 (27.42)
	moderate to severe (NIHSS = 16–20)	5 (8.06)
	severe (NIHSS = 21–42)	1 (1.61)
NIHSS at discharge mean ± SD		4.69 ± 5.26
Bloodwork parameters		
LDL Cholesterol (mg/dL) mean ± SD		108.07 ± 45.83
HDL Cholesterol (mg/dL) mean ± SD		47.62 ± 14.49
Total Cholesterol (mg/dL) mean ± SD		180.32 ± 58.81
Triglycerides (mg/dL) mean ± SD		124.16 ± 77.41
Total Lipids (mg/dL) mean ± SD		611.13 ± 183.2
Hemoglobin (g/dL) mean ± SD		13.59 ± 1.73
Thrombocytes (10^9^/L) mean ± SD		228.17 ± 59.96
Alanine aminotransferase (ALAT) (U/L) mean ± SD		28.44 ± 20.19
Aspartate aminotransaminase (ASAT) (U/L) mean ± SD		29.56 ± 22.19
Urea (mg/dL) mean ± SD		41.13 ± 25.41
Creatinine (mg/dL) mean ± SD		1.23 ± 0.68
Na (mmol/L) mean ± SD		140.66 ± 3.27
Cl (mmol/L) mean ± SD		103.63 ± 12.74
K (mmol/L) mean ± SD		4.2 ± 0.52
AR (mmol/L) mean ± SD		23.59 ± 3.98

**Table 3:** ROC parameters for the serum levels of TM in patients with acute stroke and controls. NPV: negative predictive value; PPV: positive predictive value.

TM-T1	Threshold	Specificity	Sensitivity	Accuracy	NPV	PPV
All cases	8.69	1	0.986	0.993	0.986	1
After propensity matching		1	1	1	1	1

## Data Availability

Data is contained within the article and Supplementary Material.

## Supplementary Materials

The following supporting information can be downloaded at: https://www.mdpi.com/article/10.3390/cimb46010044/s1, Supplementary Figure S1. Distribution of propensity scores. Patients were matched for the following variables: presence of diabetes, blood hypertension, chronic alcohol and tobacco use. Supplementary Table S1. Demographic, clinical and pathological profiles of patients affected by acute stroke and non-affected counterparts after popensity score matching. Supplementary Table S2. Variation of TM levels at the first measurement (T1) in patients with stroke and in the control group. Supplementary Table S3. Variation of TM levels at the second measurement (T2) in patients with stroke. Supplementary Table S4. TM values analysis with the type of stroke. Supplementary Table S5. Correlation of TM values at T_1_ and T_2_ with the biochemical parameters of the AIS patients. *R: Pearson correlation coefficient.* Supplementary Figure S2. Correlation plots of TM levels at T_1_ (A) and at T_2_ (B) with biochemical parameters in AIS patients. *R: Pearson correlation coefficient*.
