# Supplementary material for: Thrombomodulin Serum Levels—A Predictable Biomarker for the Acute Onset of Ischemic Stroke
Source: Curr Issues Mol Biol. 2024 Jan 12;46(1):677–88. doi: 10.3390/cimb46010044 (PMC10813863; doi:10.3390/cimb46010044)
Supplement: Supplementary file 1 [file cimb-46-00044-s001.zip › cimb-2824106-supplementary.pdf]

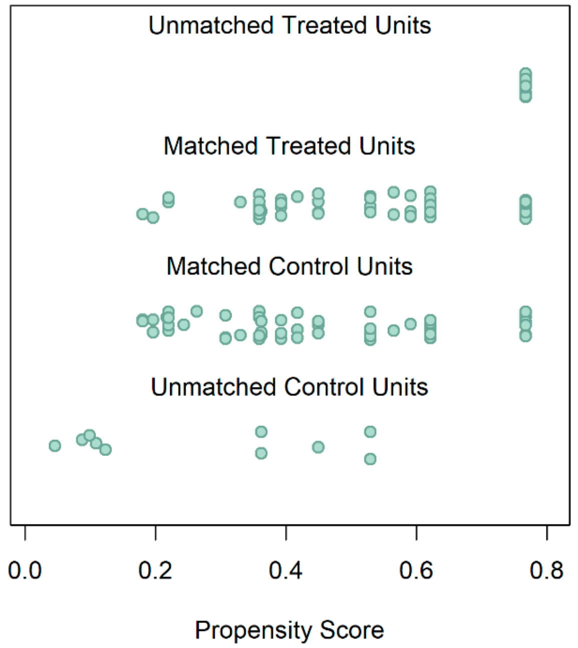

Supplementary Figure S1. Distribution of propensity scores. Patients were matched for the following variables: presence of diabetes, blood hypertension, chronic alcohol and tobacco use.

Supplementary Table S1. Demographic, clinical and pathological profiles of patients affected by acute stroke and non-affected counterparts after popensity score matching.

Variable		Control group N=58	Stroke group N=58	Chi Square p-value
**Age (years)** mean±sd		69.97±10.3	70.38±10.7	0.832
**Sex** N(%)	**Female**	24(41.38)	20(34.48)	0.444
	**Male**	34(58.62)	38(65.52)	
**Residential background** N(%)	**Urban**	29(50)	39(67.24)	0.059
	**Rural**	29(50)	19(32.76)	
**Atrial Fibrillation** N(%)	**No**	44(75.86)	36(62.07)	0.108
	**Yes**	14(24.14)	22(37.93)	
**Dyslipidemia** N(%)	**No**	29(50)	22(37.93)	0.19
	**Yes**	29(50)	36(62.07)	
**Diabetes** N(%)	**No**	37(63.79)	43(74.14)	0.229
	**Yes**	21(36.21)	15(25.86)	
**Blood Hypertension** N(%)	**grade 1**	6(10.34)	5(8.62)	0.323
	**grade 2**	31(53.45)	24(41.38)	
	**grade 3**	21(36.21)	29(50)	
**Chronic Alcohol use** N(%)	**No**	43(74.14)	48(82.76)	0.259
	**Yes**	15(25.86)	10(17.24)	
**Chronic Smoker** N(%)	**No**	41(70.69)	48(82.76)	0.124
	**Yes**	17(29.31)	10(17.24)	

Supplementary Table S2. Variation of TM levels at the first measurement (T1) in patients with stroke and in the control group.

		All cases		Control group		Stroke group	
**Variable**		**mean±sd**	**t-test p-value**	**mean±sd**	**t-test p-value**	**mean±sd**	**t-test p-value**
**Sex**	**Female**	11.72±10.22	0.126	3.8±1.88	0.511	20.53±8.25	0.053
	**Male**	14.85±12.76		3.52±1.6		24.87±9.42	
**Atrial Fibrillation** N(%)	**No**	12.99±12.09	0.411	3.51±1.69	0.289	24.23±9.05	0.236
	**Yes**	14.78±11.31		4.02±1.82		21.55±9.3	
**Dyslipidemia** N(%)	**No**	10.95±9.74	**0.021**	3.65±1.86	0.963	20.14±7.57	**0.026**
	**Yes**	15.63±12.96		3.63±1.6		25.12±9.64	
**Diabetes** N(%)	**No**	14.57±11.89	0.132	3.39±1.73	0.134	22.9±8.98	0.608
	**Yes**	11.26±11.54		4.03±1.66		24.28±10.1	
**Chronic Alcohol use** N(%)	**No**	14.54±12.1	0.071	3.72±1.76	0.586	23.01±9.71	0.685
	**Yes**	10.19±10.36		3.47±1.65		24.3±5.02	
**Chronic Smoker** N(%)	**No**	14.55±11.49	0.081	3.8±1.65	0.277	22.6±8.75	0.185
	**Yes**	10.43±12.56		3.32±1.84		26.77±11.24	

Supplementary Table S3. Variation of TM levels at the second measurement (T2) in patients with stroke.

**Variable**		**mean±sd**	**t-test p-value**
**Sex**	**Female**	19.82±6.53	**0.01**
	**Male**	25.11±8.76	
**Atrial Fibrillation** N(%)	**No**	23.35±8.53	0.744
	**Yes**	22.66±8.18	
**Dyslipidemia** N(%)	**No**	21.29±7.09	0.162
	**Yes**	24.21±8.93	
**Diabetes** N(%)	**No**	22.69±8.29	0.463
	**Yes**	24.5±8.66	
**Chronic Alcohol use** N(%)	**No**	22.97±8.79	0.783
	**Yes**	23.77±5.3	
**Chronic Smoker** N(%)	**No**	22.36±7.8	0.066
	**Yes**	27.85±10.59	

Supplementary Table S4. TM values analysis with the type of stroke

	mean±sd		
**Variable**	**Cardioembolic**	**Atherothrombotic**	**t-test p-value**
TM T1	21.55±9.3	24.23±9.05	0.236
TM T2	22.66±8.18	23.35±8.53	0.744

Supplementary Table S5. Correlation of TM values at T_1 and T_2 with the biochemical parameters of the AIS patients. *R: Pearson correlation coefficient.*

Biochemical parameter	TM T_1		TM T_2	
	R	p-value	R	p-value
LDL Cholesterol [mg/dl]	0.365	**0.002**	0.291	**0.017**
HDL Cholesterol [mg/dl]	0.14	0.253	0.064	0.608
Total Cholesterol [mg/dl]	0.333	**0.006**	0.241	**0.05**
Triglycerides [mg/dl]	0.075	0.544	0.031	0.805
Total Lipids [mg/dl]	0.255	**0.036**	0.17	0.169
Alanine aminotransferase (ALAT) [U/L]	0.415	**<0.001**	0.377	**0.002**
Aspartate aminotransaminase (ASAT) [U/L]	0.471	**<0.001**	0.475	**<0.001**
Urea [mg/dl]	-0.114	0.345	-0.006	0.961
Creatinine [mg/dl]	-0.109	0.371	0.032	0.795
Na [mmol/L]	-0.069	0.573	-0.026	0.832
Cl [mmol/L]	0.166	0.169	0.166	0.176
K [mmol/L]	-0.196	0.104	-0.176	0.151
AR [mmol/L]	-0.09	0.458	-0.063	0.607

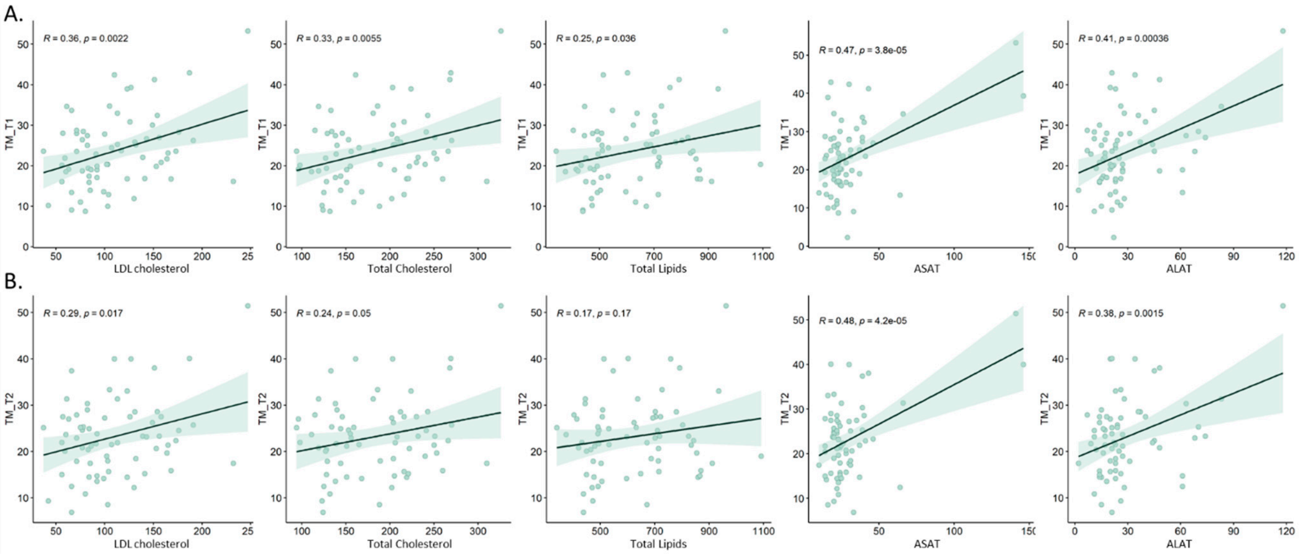

Supplementary Figure S2. Correlation plots of TM levels at T_1 (**A**) and at T_2 (**B**) with biochemical parameters in AIS patients. *R: Pearson correlation coefficient.*
